# Experimental Study of a Representative Sample to Determine the Chemical Composition of Cast Iron

**DOI:** 10.3390/ma17061255

**Published:** 2024-03-08

**Authors:** Martina Laubertova, Silvia Ruzickova, Jarmila Trpcevska, Jaroslav Briancin

**Affiliations:** 1 Institute of Recycling Technologies, Faculty of Materials, Metallurgy and Recycling, Technical University of Košice, Letná 1/9, 04 200 Košice-Sever, Slovakia; silvia.ruzickova@tuke.sk (S.R.); jarmila.trpcevska@tuke.sk (J.T.); 2 Institute of Geotechnics Slovak Academic Science, Watsonova 45, 04 001 Košice, Slovakia; briancin@saske.sk

**Keywords:** pyrometallurgy, casting, technological innovations, assaying processes, immersion sampling

## Abstract

In metallurgical practice, the material is considered of adequate quality if it meets the customer’s expectations. It is necessary to take representative samples and perform quality testing to avoid financial and intangible losses. Sample contamination and matrix and surface quality play a significant role in the accuracy of chemical analyses. The purpose of this paper is to point out the advantages of specific methods of taking samples, such as immersion and spoon sampling of molten metal, and, in the experimental part, to assess the impacts of factors affecting the quality of the sampling. The influence of time of final sampling on determining the true amount of magnesium during a single melt and the influence of duration of mixing of molten cast iron on the accuracy of chemical analysis of the control sample were investigated. It is important that the time between the modification and casting of the liquid cast iron from the casting ladle be as short as possible. This is because the magnesium burns out and thus the chemical analysis of the sample taken is not accurate. Another important factor is ensuring the melt before sampling is homogenized and has the minimum prescribed temperature (1420 °C). Increasing sample collection time will cause changes in its chemical composition.

## 1. Introduction

The chemical analysis of metal samples in the steel industry has experienced a renaissance in recent decades. Improvements have resulted in more accurate analysis, shorter turnaround times (TATs), lower costs, and more reliable results processing. There are many different techniques used to determine the composition of the alloy, but in technical practice, two are generally recommended and most often applied: X-ray fluorescence (XRF) spectroscopy and optical emission spectroscopy (OES) [1,2,3]. Optical emission spectroscopy or OES analysis is a fast analytical method designed to determine the elemental composition of various metals and alloys. Optical emission spectrometers are now more efficient, user-friendly, and precise. Foundries, which have already started profiting from the improvement in OES technology, can carry out the more accurate analyses required by stricter customer specifications for chemical analysis and make progress in their continuing efforts to acquire ISO 9000 and QS 9000 certification [4,5]. 

The capability of this technology to provide accurate analyses depends primarily on the use of correct procedures in taking samples of molten metal intended for analysis [6,7]. 

Metallurgical sampling is a fundamental part of monitoring the processing of input materials and the production of metal itself. The result of the correct chemical composition of input and output materials in the process of metal production is important for the proper course of the technological flow of material from both economic and ecological points of view. Material balances are regularly drawn up in every production plant, setting out the amount of metal obtained against the amount of metal lost in waste products, the breakdown of desirable and damaging inclusions, and losses in individual sections of the production process. Special attention must be paid to the preparation of samples taken directly from the molten metal. The turnaround time of sampling is particularly important because it enables the production technology to be controlled more flexibly [8,9,10].

In grey iron, graphite is in form of flakes. Spheroidal graphite iron is also known as ductile iron, which is obtained from grey iron by Mg treatment and inoculation. Ductile iron has good mechanical properties as compared to grey iron, such as excellent castability, superior damping capacity, and a good combination of strength, ductility, and toughness. Ductile iron is used in the automotive industry and in mechanical power transmission equipment. One of the significant contributions of this study is to point out the correct sampling of a representative sample of liquid metal for the correct preservation of the characteristics of the cast iron [11].

The incorrect taking of samples during the production process itself may cause subsequent complications in further processing. Sampling must be sophisticated and reliable because it influences the technical and economic characteristics of the production process. Several further steps follow the taking of the primary sample, namely the preparation of samples in the plant, the transfer of samples to the laboratory, communication between the plant and laboratory, automatic handling, and the processing of specimens in the laboratory, ending with final specimens prepared for analysis [12]. 

There are three main aspects in measuring the purity of metals: chemical analysis, metallographic evaluation, and techniques based on physical principles [13]. In the metal industry, it is often desirable to receive identical metal parts at different stages of production so that the quality of the final product can be precisely controlled. For example, analysis of a sample of molten metal taken from an operating furnace can be used to determine the additions to the mixture needed to produce the desired grade and composition of metal [14]. 

Sampling systems such as immersion, suction, and stream samplers are available in disc, oval, dual-thickness, reversed-dual-thickness, pin, and cylinder formats. These samplers provide highly representative sampling by improving homogeneity and eliminating dilution and contamination effects. They are recommended when ultra-high analytical accuracy and sampling yield are required. Simple to apply, easy to strip, and representative of the melt are the characteristics of a good sampler [15,16]. 

The authors investigated sampling procedures using spoon sampling and an immersion sampler. The main problems encountered with the steel samples were pinholes due to inadequate deoxidation and slag inclusions on the surface [17]. 

The advantages of taking samples directly from molten metal are shorter turnaround time, reduced complication of sampling work through simplification, and the improved potential for preparing samples suitable for chemical analysis. Samples are taken from molten metal using a spoon (spoon sampling) or lances (immersion sampling) according to the current International Standard, Steel and iron—Sampling and preparation of samples for the determination of chemical composition. Pneumatic tube posting is used for the rapid transfer of samples. Their distribution to various preparation centers and induction to the laboratory are handled automatically. Sampling molten metal by immersing the ingot mold into the melt for determination of hydrogen excludes the subjective factors affecting the sampling and enables the taking of samples from metallurgical containers at different depths [18,19,20]. 

It is important to ensure the reproductive standard of the method of sample preparation, to achieve the minimum possible number of systemic as well as random analytical errors [21,22,23]. 

For the characterization (quantitative or qualitative analysis) of the prepared alloy, it is extremely important to prepare the samples according to the requirements of the characterization method. For example, in optical microscopy, samples must be ground, polished, and etched; however, thermal analysis does not require polishing or etching. The sampling and assaying processes are continually innovated in close collaboration with the suppliers; most of the technologies applied are developed “in-house” [24,25]. 

Sample processing is an essential part of accurate sample analysis. It includes recovery of the target contaminant from the sample, cleanup to remove potential interferents (e.g., debris, non-target contaminants), grinding, and polishing. All these steps allow for accurate, highly sensitive, and reliable testing/analysis results [19,24]. 

Analytical quality control must follow the requirements of the production process, in particular, round-the-clock analytical service (24 h per day, every day of the week), the shortest possible response time (from entering the sample into the system to delivering the results), and high accuracy and reliability of sampling and analysis [8]. 

Foundry sample tools produced from advanced ceramic materials are available for sampling molten metals. Other ceramic tools for working with molten metal include small dip samplers with integral handles, stirrers designed to produce more homogeneous alloys, plain and paddle-bladed rods, and scrapers for removing slag [26,27,28].

Samples are suitable for the quick analysis of carbon, manganese, sulfur, phosphorus, and other chemical ingredients. When a sampler is inserted into the hot metal liquid, under static pressure, the hot metal liquid breaks through the slag protective layer, moving through the sampler inlet into the sampling chamber automatically. Then, the alloying element content in the cast iron can be analyzed by spectral analysis [29]. 

Emphasis is placed on the quality of samples from both chemical and physical points of view. The significance of taking and preparing representative samples is crucial for chemical analysis. The influence of time of final sampling on determining the real amount of magnesium during a single melt, the impact of duration of homogenization of molten cast iron on the accuracy of chemical analysis of the control sample, and inter-laboratory comparison of the results of chemical analyses were investigated. The purpose of the work is to describe the impacts of various factors affecting the quality of samples taken from individual cast-iron castings by applying suitable sampling methods. 

## 2. Materials and Methods

**Figure 1:**
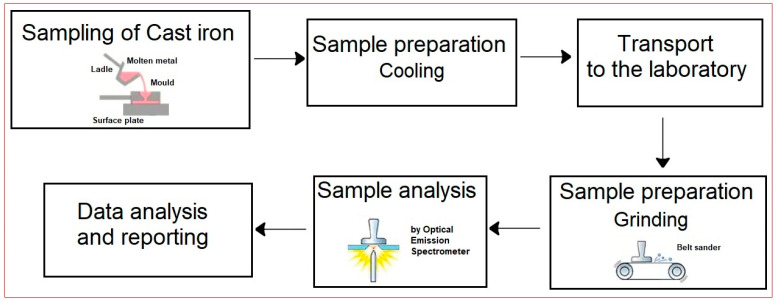
Flowchart of sample processing of molten metal and analysis at The Foundry 1 plant.

This research was carried out in cooperation with the company EUROCAST Kosice s.r.o. in Kosice, Slovakia, Europe, a subsidiary of the German foundry group Silbitz Guss GmbH. The Kosice foundry is primarily involved in producing castings made from grey cast iron, spheroidal cast iron, and steel. All the experimental work presented in this study took place during the actual casting production process at The Foundry 1 plant. The steps after taking the primary sample, such as “in-house” sample preparation, sample transport to the laboratory, information between the company and the laboratory, and automatic sample handling in the laboratory and its processing, leading to the final sample ready for analysis, are shown in the flowchart in Figure 1.

All raw materials to produce cast-iron castings are distributed in the supply hall of EUROCAST Kosice according to their chemical composition. Scrap, pig iron, coke, and returnable and other materials are located here. The carbon, sulfur, manganese, phosphorus, and silicon content of the feedstock is determined. Some elements are intentionally added to cast iron for the purpose of attaining certain specific properties and characteristics, such as copper, nickel, chromium, tin, and molybdenum. These elements subsequently cause advantageous and disadvantageous properties in cast iron. For this reason, manufacturers place increased emphasis on the absolute precision of carbon, sulfur, manganese, phosphorus, and silicon determination in the production process, from the feed materials to the finished casting. This means that the sampling basis for preparing samples is already in the supply hall. This is where the first sampling phase begins. The imported scrap is sampled. Samples are taken from the bins and the wagons, with four samples taken from each heap. The obtained samples are labeled and subsequently subjected to chemical analysis in the laboratory.

**Figure 2:**
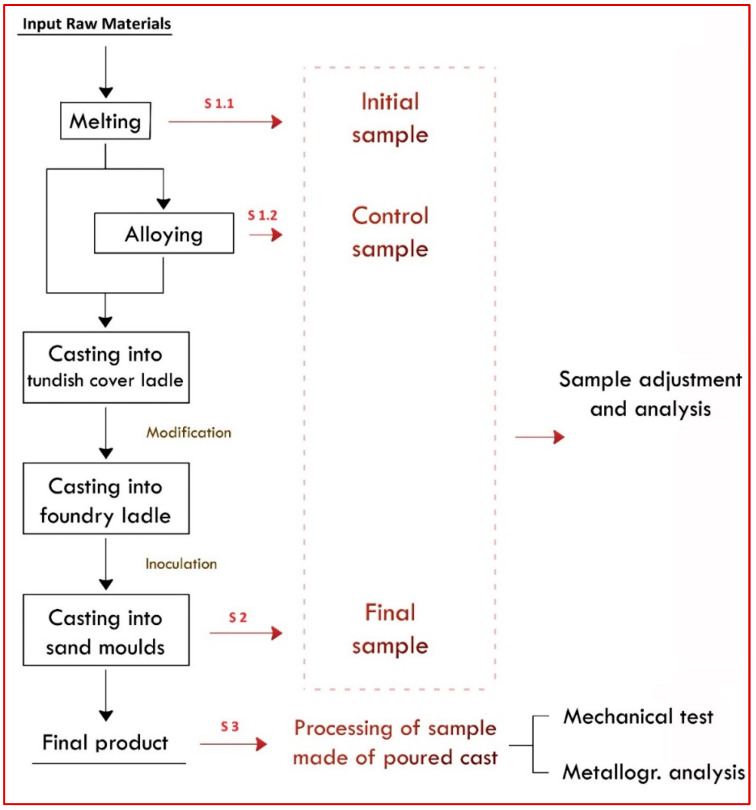
Flowchart showing the taking and preparing of samples at The Foundry 1 plant.

The melting is carried out in two 6-ton JUNKER induction furnaces, in which the metal is melted alternately (one furnace melts, the other casts). Raw materials are fed in from the supply hall and used to produce liquid metal (grey and ductile iron). Figure 2 presents a flowchart of the casting production from molten metal, indicating the sampling points. The furnaces are charged with input materials from the supply hall, and they produce molten metal in the form of grey and spheroidal cast iron. 

Three main types of samples are taken in this operation: S 1.1 = Initial or starting sample, S 1.2 = Control sample (correcting sample), S 2 = Final sample, and S 3 = In-cast sample (trial block). The in-cast sample is made of poured cast and taken from the final product after cooling. This sample is subjected to a turning process by a lathe machining operation. This in-cast sample is intended for establishing the mechanical properties of the cast material. Test bars and prisms are made from this in-cast sample by cutting and mechanical machining. These bars and prisms are used to verify the intended mechanical properties using tensile tests, impact tests, and hardness tests. A sample for a metallographic examination to confirm graphite structure by mechanical processing (turning) is also obtained according to current European Standards (Founding—Spheroidal graphite cast irons, EN 1563:2011) [30]. 

**Figure 3:**
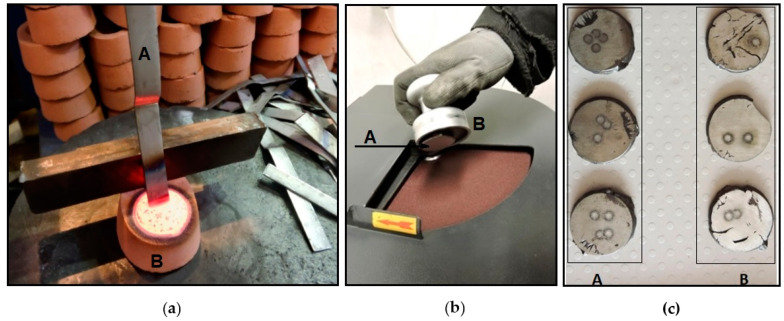
Preparation of sample for chemical analysis. (**a**) Solidification of starting sample in a quanto pot (S 1.1): silica sand quanto pots—A; steel bar for taking slag—B. (**b**) Grinding sample: disc sample—A; magnetic sample holder—B. (**c**) Correct samples—A; incorrect samples—B.

Figure 3 shows the solidification of the starting sample as well as its grinding and polishing prior to the chemical analysis using optical emission spectroscopy.

The starting sample was taken from the furnace before casting. First, the slag was skimmed from the surface of the molten metal. Before the sample was taken, it was necessary to clean the sampling sibral ladle in the molten metal itself. The sample was drawn into a quanto pot (sand-lined container) (Figure 3a) and subsequently cooled, first in air and then in water. 

The quality of the sample is essential; there can be the presence of defects and oxides on the surfaces of the disk sample, the surface layers of the disk may be subject to segregation, and the central portion may be porous and subject to shrinkage or other thermal effects. Grinding is necessary to ensure that the procedures for preparing the surface of the disk for analysis by spectrochemical methods expose a layer of metal that is representative of the chemical composition of the sample. 

It is important to remove a 1 mm to 2 mm layer from the surface of a disk sample obtained from liquid metal to expose a part of the sample that is suitable for the analysis method selected. A magnetic sample holder is one that holds the sample when grinding (Figure 3b). If its chemical composition proves to be correct, then the molten metal is poured into the casting ladle, or the tundish ladle for alloying, and then another sample is taken (control sample). After pouring into the tundish ladle, the grey cast iron is modified by the use of magnesium (Mg), producing spheroidal shapes; then, when it is poured into the casting ladle, it undergoes inoculation.

Melting of the input materials was done using an Induction Melting Furnace (MFT Ge 6000 Junker, OTTO Junker GmbH, Simmerath, Germany, 1993) with a melting capacity of 5350 kg/h, connection power of 2.9 MW, at 500 Hz, and with weighting equipment, an inverter, and a transformer water cooling system. In these experiments, we selected samples from cast iron 20-0400 (also known as EN-GJS 400-15); their chemical compositions are listed in Table 1, Table 2 and Table 3. The Initial sample is taken from the furnace before casting (Table 1). The Control sample is taken after alloying (Table 2). After casting in the intermediate ladle, the gray cast iron is modified with Mg and made into a block. The Final sample is taken after the cast iron is in the molds (Table 3). 

Chemical analysis was determined by a Thermo ARL Optical Emission Spectrometer (Thermo ARL, Dreieich, Germany, 1982). Each sample was ground in a sample grinding machine (HT 350-2 Herzog, Osnabrück, Germany, 1991). Samples were subjected to scanning electron microscopy (SEM) analysis along with energy-dispersive spectrometry using MIRA3 FE-SEM (resolution: 1.2 nm at 30 kV; 2.3 nm at 3 kV, TESCAN, Brno, Czech Republic).

**Figure 4:**
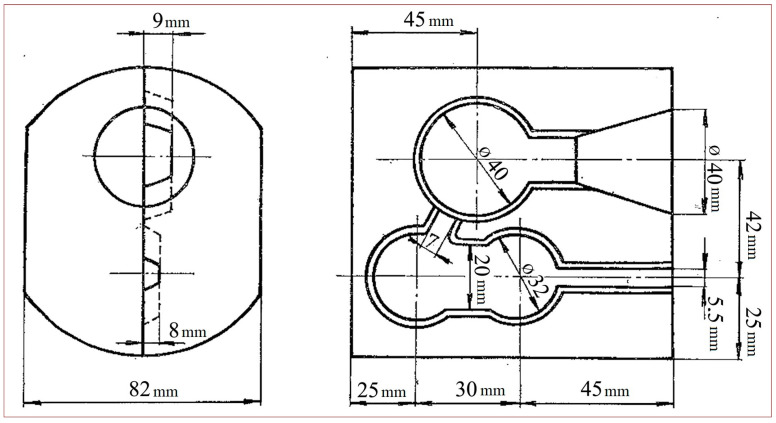
Metal sample molds produce samples for arc-spark spectrometers: Scheme of molds [12].

The final sample was taken after the molten metal was poured into the molds. Two samples were taken, namely from the penultimate or the last mold while the metal was still molten. Samples were transferred into an ingot mold using spoon sampling, and they were taken together to the two-piece copper mold (Figure 4). The two-piece copper mold is a simple but effective tool that can be reused, over and over, to produce metal discs for fast and easy chemical testing [31]. 

**Figure 5:**
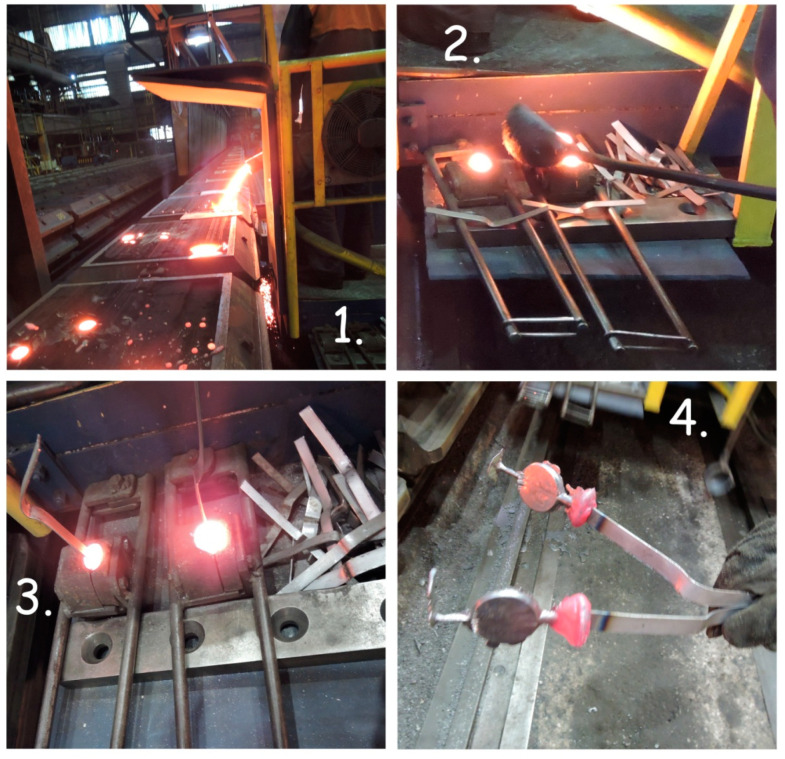
The final sample-taking process from the last foundry mold: 1—taking sample; 2—casting sample to the copper sample mold; 3—cooling sample; 4—final sample (S 2).

Figure 5 shows the final sample (S 2), taking the process from the last mold.

**Figure 6:**
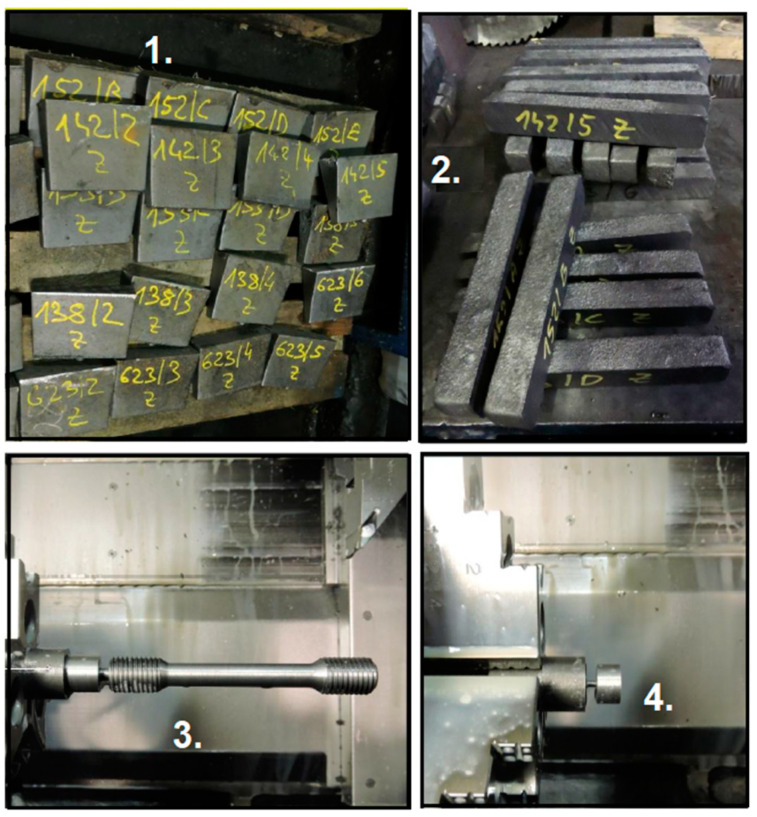
S 3 in-cast samples: 1—trial blocks; 2—rods cut out of blocks; 3—testing bar for mechanical properties; 4—a sample to determine the structure of the material.

The next section on this line is the cooling unit, where every casting cools down freely. In the case of mold castings, trial blocks (S 3) are also cast, resulting in in-cast samples, which are intended for establishing the mechanical properties of the cast material. Two trial blocks are cast from one ladle, in case one of them is faulty in some way (e.g., crumbling or porous). These blocks are subjected to cutting and turning to produce testing rods and bars, which are taken at least once every day to the central laboratory for mechanical testing and sampling to determine the structure of the material (Figure 6). 

Finished, de-burred castings are stored in the shipping hall and prepared for dispatching to customers. The crystallization process of spheroidal graphite cast iron is significantly influenced by the chilling (solidification) rate. Rapid chilling produces small nodules. It is important to take the final sample from the last of the molds in order to determine the chemical composition and the true amount of magnesium in the cast iron [32]. 

The magnesium content dwindles with time, as it burns off. In the air, magnesium becomes covered in a layer of oxide, which passivates it. It has a great affinity to oxygen and sulfur. It begins to burn off when added to the mixing ladle. This happens due to the bound oxygen, which promotes the burning. Its ignition temperature is just 250 °C. The course of the reaction between magnesium and oxygen is characterized by radiation of very intensive light and great heat, which may reach temperatures up to 2200 °C (Reaction 1) [33,34].

2Mg_(s)_ + O_2(g)_ → 2MgO_(s)_ (white powder) + energy (1)

The content of magnesium added to the grey cast iron melt to bring about its modification was investigated. Its content in the final casting varied between 0.03 and 0.05%. The amount of residual magnesium required to produce spheroidal graphite depends on factors such as the duration of casting and chilling and the temperature of casting. Once the molten cast iron is poured into the mold, it starts to solidify. 

The duration of solidification depends on the mass of the given casting. The larger the casting, the longer the metal takes to solidify; thus, more magnesium needs to be added for modification. Another factor is the temperature at which the molten cast iron is cast. If the casting temperature reaches 1500 °C, Mg burns off immediately. The ideal temperature of casting is in the range 1375–1395 °C. If the tapping temperature is higher, e.g., 1420 °C, then it is necessary to add more Mg. The required amount of magnesium depends not only on the proportion of it in the modifier but also on the granularity of the cast iron. 

## 3. Results and Discussion

### 3.1. Influence of Time of Final Sampling on Determining the True Amount of Magnesium during a Single Melt

The amount of Mg that needs to be added to the cast iron is a function of its original level of sulfur; the higher the original sulfur content, the more Mg that must be added for the modification (every 1% of desulfurization consumes 0.75% Mg). For this reason, the original metal in the melt should contain as little sulfur as possible. A low residual level of Mg means an insufficient amount was used for effective spheroidization. Conversely, a high residual level has an unfavorable effect on the cast iron’s mechanical properties, and excessive Mg increases the formation of carbides as well. 

The first experiment was designed with these factors in mind. Final samples were taken from the first and the last mold to demonstrate the amount of magnesium that burns off with time. The results of the chemical analysis for this factor are presented in Table 4, and the prescribed chemical composition for the given material from the final chemical analysis is set out in Table 5. Designation of the model No., e.g., D 1527/1, means a request from the customer. Designation of the material, e.g., 20-0431 (also known as GJS-400-18-LT), represents the internal designation of the material produced in the foundry. The assigned chemical composition and melting charge correspond to this material number. 

**Figure 7:**
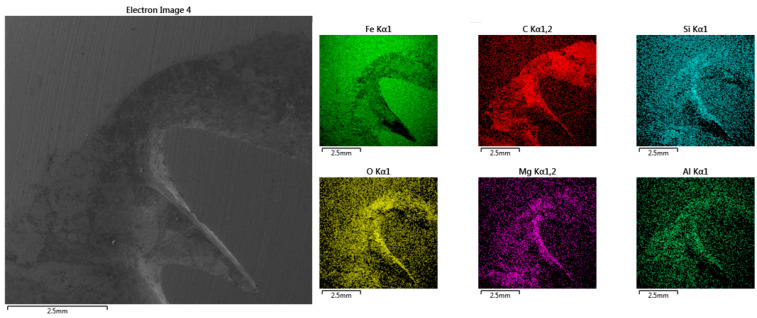
Results of EDX map surface analysis.

Specimens intended for chemical analysis (prepared from starting, checking, and final samples) should have a minimum number of defects, e.g., contractions or sharp edges, that could damage the spectrometer; see Figure 3c. Larger amounts of shrinkage can distort the outcome of the analysis, as demonstrated by the results of EDX map surface analysis presented in Figure 7, where it is evident that the contractions led to the accumulation of certain elements, such as C, Si, O, Mg, and Al. In this case, the sampling must be repeated, extending the turnaround period. 

Assessment of Influence 1

It was found that the interval between the modification and casting of molten metal from the casting ladle should be as short as possible, since Mg burns off with time and at higher temperatures (over 1300 °C) in the ladle. This is confirmed by the chemical analysis presented in [24]. Casting into molds in smaller numbers but with greater weights takes roughly as much time as casting into a larger number of molds with lower weights because it takes longer to pour heavier castings than those with lower weights.

The content of Mg was higher in samples taken from the first molds than in those from the last. The final samples must be taken from the last or penultimate mold to monitor and determine the true values of Mg in the cast iron. The loss of magnesium by burning in the metal depends on the following conditions: the type of modifier used, the tapping temperature in the furnace (the higher the temperature, the greater the burnout), the casting temperature (also the higher the temperature, the greater the burnout), the cleanliness of the casting and processing ladles, the number of cast forms, and the casting temperature. The magnesium content decreases with time, i.e., the larger the number of molds, the longer the casting will be.

### 3.2. Impact of Duration of Mixing of Molten Cast Iron on the Accuracy of Chemical Analysis of the Control Sample

Concerning this factor, we investigated the change in the chemical composition of samples taken after the alloying of the molten cast iron. In this experiment, the following input materials were used as alloying additives: 17 kg quartz, 2.5 kg manganese, 17 kg copper, 400 kg returns, and 100 kg steel scrap. The alloying additives were kept separate from each other in color-coded buckets. The metal gradually melted in the furnace with continuous mixing. Some of the additives melted almost immediately, while others took longer to melt due to their having higher melting points. Copper, for example, has a melting temperature of 1083 °C, and manganese, 1245 °C.

In between the first (starting) sample and the control sample (taken after alloying), three other samples were taken. These were taken at intervals of 20 s, and before each one, the temperature of the melt was measured and recorded as shown in Table 6. This experiment also monitored the influence on the results of sampling when there was insufficient mixing during alloying. The prescribed chemical composition for material 20-0505 (known also “EN-GJS 500-7—Knorr”) is as follows: 3.73–3.83 wt.% C and 1.4–1.5 wt.% Si (Table 7).

Assessment of influence 2

The results of the chemical analysis shown in Table 6 indicate that the samples taken just after alloying of the molten cast iron (each at an interval of 20 s) were affected by the insufficient time allowed for the alloying additives to melt and return material. Each sample must be homogenized before sampling. There was an evident drop in the temperature of the melt after the final charging and alloying with additives, from 1420 °C to 1281 °C. However, after 120 s, while the checking sample was taken, the temperature rose almost to its original level. This can also be seen in the content of manganese, whose melting temperature is 1245 °C. When the melt temperature drops after alloying and thus approaches the melting point of manganese, its solubility is reduced as well. Once the checking sample is taken, the chemical composition of the melt becomes stabilized, since the alloying additives in the induction furnace melt over time and are mixed in.

The suitable period for taking the checking sample depends on the amount and type of material added into the furnace during alloying. After alloying is the time for measuring the temperature of the melt in the furnace. The sample is taken only after the complete melting of all additives and charging materials, at a minimum temperature of at least 1420 °C. The sample is taken immediately once this temperature is reached. If there is a longer delay in the sampling, a change in the chemical composition occurs.

### 3.3. Inter-Laboratory Comparison of the Results of Chemical Analysis

To find the factors influencing the chemical analysis, we compared the results of the separate chemical analyses of two companies, Foundry A (Foundry in Slovakia) and Foundry B (EUROCAST Kosice). Before using the spectrometer for the chemical analysis, itself, it is necessary to calibrate the instruments. In Foundry B, the calibration was achieved using reference samples (SUS_A6, STAN). The maximum deviations occurring during calibration were 5% (Foundry A) and 1.19 wt.% (Foundry B). The certified reference sample compositions, according to which the coefficient for Foundry B was calculated, were as follows: 3.22 wt.% C; 2.53 wt.% Si; 0.7 wt.% Mn; 0.036 wt.% P; 0.007 wt.% S. For Foundry A, they were 3.6 wt.% C; 2.7 wt.% Si; 1.1 wt.% Mn; 0.2 wt.% P; and 0.01 wt.% S. The actual measured sample compositions were multiplied by these coefficients. There must be a constant temperature in the room containing the spectrometer. If it varies during the day, the instrument must be calibrated again.

The experimental procedure was as follows: initially, five samples were taken in the foundry of EUROCAST Kosice. They were taken from the furnace after alloying at a temperature of 1500 °C. The prescribed chemical composition for material 20-0434 is as follows: C = 3.75–3.85 wt.% and Si = 1.35–1.45 wt.%. Four measurements were carried out on each sample using the spectrometer, from which the average values were calculated. The values from both plants are presented in Table 8, and their statistical evaluation is presented in Table 9.

Assessment of influence 3

The inter-laboratory comparison revealed that various factors affect the chemical analysis of samples. The most important factor is the reference sample used to calibrate the given spectrometer. All laboratory measurements should be performed on spectrometers calibrated using the same reference sample; otherwise, the results of the same analyses may differ, as can be seen in the results of our experiment. It is necessary, moreover, that the reference sample have a composition of elements as similar as possible to that which we want to achieve in production; otherwise, inaccuracies can occur. Another factor is the ambient temperature. In most laboratories, the temperature is maintained at the same level by using air conditioning.

The final but no less significant factor is grinding. During analysis with various spectrometers, specimens need to be perfectly ground and polished. If the instrument picks up an irregularity on an imperfectly polished surface, this can distort the results of the analysis, rendering them unrepresentative and unusable.

## 4. Conclusions

From the results obtained in this experimental study, it is possible to assess the impact of the timing of sampling on the results of the chemical analysis of cast iron in the studied samples in the furnace and in the molds, as follows:

– The sample from the furnace must be taken immediately when a temperature of at least 1420 °C is reached, after the complete melting of all additives and charging materials; this means that the hot metal is properly homogenized and the alloying additions are fully dissolved.
– For desulfurization, 1% of sulfur is consumed by 0.75% magnesium. The content of magnesium in the sample taken from the first mold sample was 0.046 wt.%, with 0.023 wt% sulfur. The content of magnesium in the sample taken from the last form was 0.038 wt.%, with 0.007 wt.% S. The contents of Cu, P, and Mn did not change. It was confirmed that the factors affecting the burning-off rate are the duration and temperature of the molten metal in the ladle.
– For accurate analysis with various spectrometers in different foundries, specimens must, in any case, have perfectly ground and polished surfaces, and the temperature in the spectrometer room must be constant and maintained during the analysis using air conditioning.
– Both sides of the casting business, the producer and consumer, have a clear interest in gaining accurate testing results. If this does not happen, the future consequence could be complications in the form of arbitration disputes or refusal to sign further contracts, outcomes which need to be and can be prevented.

## Figures and Tables

**Table 1:** Chemical analysis of the Initial sample.

	Base Elements (wt.%)						
Material	C	Si	Mn	P	S	Mg	Cu
20-0400	3.870	1.131	0.200	0.022	0.009	0.001	0.024

**Table 2:** Chemical analysis of the Control sample.

	Base Elements (wt.%)						
Material	C	Si	Mn	P	S	Mg	Cu
20-0400	3.860	1.217	0.204	0.022	0.011	0.001	0.024

**Table 3:** Chemical analysis of the Final sample.

	Base Elements (wt.%)						
Material	C	Si	Mn	P	S	Mg	Cu
20-0400	3.700	2.279	0.215	0.022	0.007	0.047	0.026

**Table 4:** Results of chemical analysis for factor 1.

Sample No.	Model No.	T (°C)	Material	Shape No.	Sampling Shape	Base Elements (wt.%)						
						C	Si	Mn	P	S	Mg	Cu
1	D 1527/1	1385	20-0431	6	1/1	3.7	2.27	0.25	0.023	0.008	0.046	0.04
					1/6	3.69	2.28	0.25	0.023	0.007	0.038	0.04
2	D 1527/1	1385	20-0431	6	2/1	3.77	2.28	0.25	0.023	0.007	0.044	0.04
					2/6	3.76	2.29	0.25	0.022	0.007	0.035	0.04
3	D 1527/1	1385	20-0431	6	3/1	3.72	2.27	0.25	0.022	0.006	0.042	0.04
					3/6	3.66	2.26	0.24	0.022	0.008	0.035	0.04
4	A5E0941302	1430	20-0400	8	4/1	3.72	2.22	0.19	0.023	0.007	0.041	0.03
					4/8	3.74	2.21	0.19	0.023	0.007	0.036	0.03
5	A5E0941302	1430	20-0400	8	5/1	3.73	2.27	0.19	0.024	0.007	0.042	0.03
					5/8	3.72	2.25	0.19	0.023	0.007	0.036	0.03
6	10170272	1390	20-0400	12	6/1	3.68	2.24	0.19	0.022	0.007	0.044	0.03
					6/12	3.68	2.24	0.19	0.022	0.007	0.035	0.03
7	10170272	1390	20-0400	12	7/1	3.69	2.24	0.19	0.023	0.007	0.043	0.03
					7/12	3.69	2.23	0.19	0.022	0.007	0.036	0.03
8	10170272	1390	20-0400	15	8/1	3.65	2.23	0.19	0.023	0.007	0.045	0.03
					8/15	3.64	2.23	0.19	0.022	0.007	0.038	0.03

**Table 5:** Prescribed chemical composition for the material 20-0400.

	Base Elements (wt.%)						
Material	C	Si	Mn	P	S	Mg	Cu
20-0400	3.6–3.7	2.2–2.3	max 0.25	max 0.05	max 0.01	0.035–0.048	max 0.027

**Table 6:** Results of chemical analysis for factor 2.

Sample No.	Type of Sample	Material	Temperature * (°C)	Time ** (s)	Base Elements (wt.%)			
					C	Si	Mn	Cu
1	Initial sample S 1.1	20-0505	1420	-	3.920	1.166	0.237	0.088
2	Experiment 1	20-0505	1281	20	3.770	1.670	0.245	0.277
3	Experiment 2	20-0505	1300	40	3.820	1.448	0.276	0.385
4	Experiment 3	20-0505	1320	60	3.840	1.378	0.272	0.310
5	Control sample S 1.2	20-0505	1400	120	3.800	1.360	0.263	0.365

* Temperature of melt during sample taking. ** Time interval after alloying of the molten cast iron.

**Table 7:** Prescribed chemical composition for the material 20-0505.

	Base Elements (wt.%)	
Material	C	Si
20-0505	3.73–3.83	1.4–1.5

**Table 8:** Results of chemical analysis for inter-laboratory comparison.

Company	Spectrometer	Sample No.	Chemical Analysis (wt.%)				
			C	Si	Mn	P	S
Foundry A	BAIRD DV4	1	3.81	1.525	0.25	0.03	0.007
		2	3.78	1.560	0.26	0.03	0.006
		3	3.86	1.630	0.27	0.03	0.006
		4	3.86	1.640	0.27	0.04	0.007
		5	3.88	1.670	0.27	0.04	0.008
		Mean	3.838	1.605	0.264	0.034	0.007
Foundry B	ARL 3460	1	3.82	1.394	0.235	0.024	0.012
		2	3.84	1.391	0.236	0.023	0.012
		3	3.84	1.394	0.236	0.023	0.012
		4	3.82	1.396	0.236	0.023	0.012
		5	3.85	1.394	0.237	0.023	0.013
		Mean	3.834	1.394	0.236	0.023	0.012

**Table 9:** Statistical characteristics for inter-laboratory comparison.

Statistical Characteristics	Foundry A		Foundry B	
	C	Si	C	Si
Mean	3.838	1.605	3.834	1.394
Standard deviation	0.037	0.0538	0.012	0.0016
Variance	0.0013	0.0029	0.0001	2.6 × 10^−6^
Median	3.86	1.63	3.84	1.394

## Data Availability

Data are contained within the article.
